# A Model of Synaptic Reconsolidation

**DOI:** 10.3389/fnins.2016.00206

**Published:** 2016-05-18

**Authors:** David B. Kastner, Tilo Schwalger, Lorric Ziegler, Wulfram Gerstner

**Affiliations:** School of Computer and Communication Sciences and Brain Mind Institute, School of Life Sciences, Ecole Polytechnique Fédérale de LausanneLausanne, Switzerland

**Keywords:** reconsolidation, synaptic plasticity, neuron modeling, reduced model, memory dynamics

## Abstract

Reconsolidation of memories has mostly been studied at the behavioral and molecular level. Here, we put forward a simple extension of existing computational models of synaptic consolidation to capture hippocampal slice experiments that have been interpreted as reconsolidation at the synaptic level. The model implements reconsolidation through stabilization of consolidated synapses by stabilizing entities combined with an activity-dependent reservoir of stabilizing entities that are immune to protein synthesis inhibition (PSI). We derive a reduced version of our model to explore the conditions under which synaptic reconsolidation does or does not occur, often referred to as the boundary conditions of reconsolidation. We find that our computational model of synaptic reconsolidation displays complex boundary conditions. Our results suggest that a limited resource of hypothetical stabilizing molecules or complexes, which may be implemented by protein phosphorylation or different receptor subtypes, can underlie the phenomenon of synaptic reconsolidation.

## Introduction

Reconsolidation describes a process for the alteration of memories, and highlights the dynamic nature of information processing and storage in the brain. Reconsolidation represents the phenomenon that recently triggered memories are susceptible to degradation whereas memories that have not been retrieved are spared from degradation (Nader et al., 2000). Research has shown reconsolidation to be a general phenomenon, occurring in a wide array of species, including humans (Kroes et al., 2014), and a diverse set of brain regions (Besnard et al., 2012), and has exposed various synaptic and molecular components underlying reconsolidation (Tronson and Taylor, 2007). However, scant evidence exists as to the nature of the circuit and cellular level phenomenon of reconsolidation, as opposed to the phenomenon of consolidation—the initial storage of a memory—for which a vast literature exists based on slice electrophysiology (Redondo and Morris, 2011).

Recent reviews (Nader and Hardt, 2009; Besnard et al., 2012) have highlighted the potential link of behavioral reconsolidation to experiments on long-term potentiation (LTP) in hippocampal slices. To make that link they cited an elegant set of experiments (Fonseca et al., 2006a) that caused the degradation of potentiated synapses through the interaction of stimulation and PSI.

Although cognitive and conceptual models have elucidated functional roles for reconsolidation (Blumenfeld et al., 2006; Siegelmann, 2008; Osan et al., 2011; Nowicki et al., 2013), and a simplified molecular model has been proposed to explain some aspects of reconsolidation (Zhang et al., 2010), synaptic models of reconsolidation have been lacking. Here we extend models of LTP to capture the results of Fonseca et al. (2006a), and simulate a process akin to reconsolidation in spiking leaky integrate and fire neurons.

Computational models of consolidation involve a cascade of different processes acting on different time scales (Fusi et al., 2005). Consolidation models have been formulated at the level of transitions between abstract states (Fusi et al., 2005; Barrett et al., 2009) or as extensions to models of spike-timing dependent plasticity (Gerstner et al., 1996; Song et al., 2000) for induction of plasticity so as to include the first steps of synaptic stabilization (Brader et al., 2007; Clopath et al., 2008; Ziegler et al., 2015). The model presented here adds two features to existing models of consolidation: first, a stabilization process at each potentiated synapse, and second, an activity-dependent pool of hypothetical stabilizing entities from which all synapses within a single neuron draw upon for stabilization. The dynamics of the stabilizing entities, established by state transitions between bound and unbound states, determine when reconsolidation occurs, and form the substrate for the so-called boundary conditions characteristic of behavioral reconsolidation. A particular focus of our study has been put on the role of PSI during synaptic reconsolidation. We match our model to existing data using high-frequency stimulation (HFS) to produce LTP of synapses, and discuss the need for multiple, interacting, activity-dependent processes in memory formation.

## Materials and methods

### General setup

We consider a postsynaptic neuron that receives input from *N* presynaptic neurons. The *N* synapses between the presynaptic ensemble and the postsynaptic target are subject to synaptic plasticity and consolidation, i.e., activity-dependent changes of the synaptic efficacies and slower internal synaptic states. Specifically, we consider two models of synaptic consolidation (Barrett et al., 2009; Ziegler et al., 2015), that reproduce a range of experimental data on synaptic tagging and consolidation in the hippocampus (see “Write-protected model” and “State-based model” below), but the method could also be applied to other models (e.g., Brader et al., 2007).

In the following, we put forward a generic extension of such models that endows synapses with synaptic reconsolidation-like dynamics and yields a possible explanation for the slice experiments of Fonseca et al. (2006a). Toward that end, we exploit the fact that synaptic consolidation models work on different time scales (Fusi et al., 2005). We require that the basic synapse model that we want to extend possesses variables on at least two different time scales. First, we assume that the synapse model exhibits a variable $I_{j}^{A}$ that reflects the recent coactivity of pre- and postsynaptic neurons at synapse *j*. This activity-related variable picks up correlations between pre- and postsynaptic firing and might result from a Hebbian learning rule, such as the triplet spike-timing dependent plasticity (STDP) rule (Pfister and Gerstner, 2006) discussed below. In our stimulation protocol, $I_{j}^{A}$ reflects the presence of strong extracellular stimulation of presynaptic neurons *j* because such stimulation is known to cause an increase in the coactivation of pre- and postsynaptic neurons. Second, we assume that the slowest timescale of the synaptic model is characterized by a variable *z*_*j*_ that represents the state of consolidation at synapse *j*. We can assume that this variable is normalized such that a positive value, *z*_*j*_ > 0, signifies that the synapse is in a consolidated, “strong” state, whereas a negative value indicates that the synapse is not consolidated, or in a “weak” state. In the following, a consolidated synapse will be also referred to as a “big” synapse.

### Stabilizing entity

**Figure 1:**
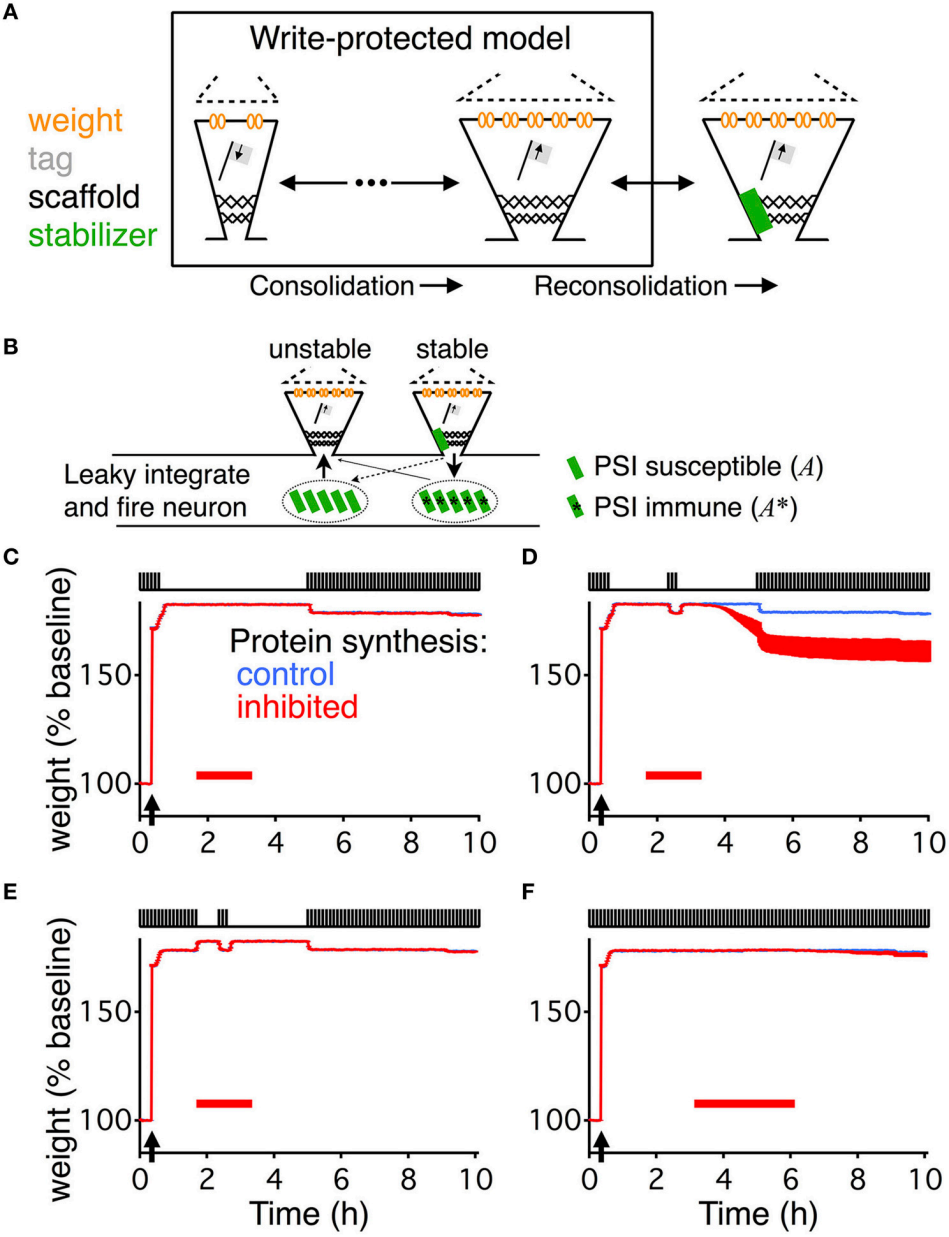
**Write-protected model extended with dynamic stabilization captures reconsolidation. (A)** The write-protected model simulates the dynamics of synapses as they transition from low weight, low tag, and small scaffold (left) to their big, consolidated state (middle). The write-protected model contains multiple interacting steps, indicated by the broken arrows linking the small synapse to the big synapse. To extend the write-protected model to capture reconsolidation we added an additional step (right), whereby a consolidated synapse binds to a stabilizing entity (green rectangle), identified as *A* and A^*^ (see Equation 2), and only when it is bound can it remain in the consolidated state. **(B)** Stabilization (destabilization) is a process in which a synapse binds (unbinds) a stabilizing entity, *A* and A^*^ (see Equation 2). An unstable big synapse (left) can bind a stabilizer coming from two different pools, a PSI susceptible pool (green rectangles, left, *A* from Equation 2), or from a PSI immune pool (green rectangles with asterisk, right, A^*^ from Equation 2). A stabilized synapse (right) is more likely to release the stabilizer into the PSI immune pool (solid arrow) than into the PSI susceptible pool. All synapses on the postsynaptic neuron, which is modeled as a leaky integrate-and-fire neuron, use the same pool of stabilizing entities. For **(C–F)** the upper part of the figure shows a cartooned version of the stimulation. All paradigms were matched to Fonseca et al. (2006a). Low-frequency stimulation, as indicated by the pulse-train on the top of each panel, was 0.1 Hz. HFS, to induce LTP, occurred where indicated by the arrow. For each panel there are two curves, one in blue (often covered by the red, most easily seen in **(D)**, which shows the response of the model without PSI, and one in red which shows the response of the model with PSI. The red bar indicates the timing and duration of the PSI. The output of the model is the average weight across all synapses, and this is averaged across ten different simulated neurons. For **(C–F)** the width of the line indicates s.e.m.

A consolidated synapse interacts with hypothetical stabilizing entities, *A*, that are synthesized in the postsynaptic cell (Figures 1A,B). The stabilizing entities *A* could be proteins or more complex compounds. These entities can bind to any unbound “big” synapse *Syn*_*j*_ (*z*_*j*_ > 0), and thereby stabilize its “big” state. The rate at which this synapse gets bound is *k*_1_*N*_*A*_*H*(*z*_*j*_), where *k*_1_ is the constant binding rate per stabilizing entity; *N*_*A*_ is the number of available entities *A* that are not yet bound to a synapse; and *H*(*x*) denotes the Heaviside step function with *H*(*x*) = 1 for *x* ≥ 0 and zero otherwise. In order to model the experimental results of Fonseca et al. (2006a), we need to specify how protein-synthesis inhibition is implemented in our model. We assume that during PSI, the synthesis of *A* is blocked and the stabilizing entities degrade rapidly compared to the relatively long time scale on which pharmacological PSI is applied. This time-scale separation essentially amounts to setting the number *N*_*A*_ of available unbound entities to zero during application of PSI.

To capture the combined effect of PSI duration and low-frequency stimulation (LFS) reported in Fonseca et al. (2006a), we posit activity-dependent unbinding of stabilizing entities from stable synapses. After unbinding, the stabilizing entity can exist in one of two different forms: the original form *A*, characterized by a high rate of degradation, and an immune form A^*^, which does not degrade on the time scale during which PSI is applied. In this way, the pool of immune entities A^*^ is resistant against PSI, and forms an effective reservoir of stabilizing entities for consolidated synapses. Like the original form, the immune form can bind to an unbound big synapse, and thereby stabilize the synapse even in the presence of PSI. The binding rate of a big synapse with A^*^ is $k_{3}N_{A^{*}}H\left(z_{j}\right)$, where *k*_3_ is a constant rate and $N_{A^{*}}$ is the number of A^*^ entities.

To capture the effect of LFS on the stability of consolidation, as reported in Fonseca et al. (2006a), we made the growth of the immune pool activity-dependent. This can be achieved by activity-dependent unbinding rates, $k_{2}^{j}$ and $k_{4}^{j}$, corresponding to the unbinding of a bound synapse into the forms *A* and A^*^, respectively. Specifically, LFS yields an increased synapse specific input, $I_{j}^{A}$, which in turn results in increased unbinding rates. This is modeled by the logistic function

(1) $$\begin{array}{l} k_{\alpha }^{j}\left(I_{j}^{A}\right)=k_{0}+\frac{m}{1+\exp\left(\frac{I_{0}^{\alpha }-I_{j}^{A}}{r}\right)}, \end{array}$$

α = 2, 4. In Equation (1), *k*_0_ is the minimal unbinding rate, *m* + *k*_0_ is the maximal unbinding rate, *r* is the rate of increase of the unbinding rate, and $I_{0}^{\alpha }$ is the input value that leads to half maximal unbinding (the value is different for $k_{2}^{j}$ and $k_{4}^{j}$). Taken together, our model for synaptic stabilization can be summarized by the following reaction scheme

(2) $$A+{\text{Syn}}_{j}\overset{H(z_{j})k_{1}}{\underset{k_{2}^{j}\left(I_{j}^{A}\right)}{⇌}}{\left(A\text{Syn}\right)}_{j}\;\overset{k_{4}^{j}\left(I_{j}^{A}\right)}{\underset{H\left(z_{j}\right)k_{3}}{⇌}}A^{*}+{\text{ Syn}}_{j}.$$

A big synapses (i.e., *z*_*j*_ > 0) will eventually decay and transition to a value *z*_*j*_ < 0 if it is not bound to a stabilizing entity, see “extended write-protected model” and “extended state-based model” below. In this way, the model can be seen as an activity-dependent creation of a PSI immune reservoir of entities A^*^ that are useful for the stabilization of consolidated synapses.

The total number of stabilizing entities available in the postsynaptic cells is $N_{A,tot}=N_{A}+N_{A^{*}}+N_{A\text{Syn}}$. In the absence of PSI, this number is constant. For simplicity, we also assume that before and after PSI this number remains the same, which in a biological system could be realized by a homeostatic mechanism. However, this assumption is not essential for the main mechanism of synaptic stabilization and creation of the reservoir. For the stochastic simulation of the *N*_*A, tot*_ stabilizers we do not need to keep track of each stabilizing entity but only the total numbers *N*_*A*_ and $N_{A^{*}}$, respectively, as well as the state of each synapse. In each time step of length Δ*t*, we compute the binding and unbinding of all synapses in a random sequence. If a synapse was unbound, we first calculate the probability $p_{j}^{\text{bind}}\;=\;\left(k_{1}N_{A}+k_{3}N_{A^{*}}\right)H\left(z_{j}\right)\Delta t$ that the synapse binds to a stabilizing entity. A binding event takes place if a uniform random number $R_{j}^{1}\in \left[0,1\right)$ is smaller than $p_{j}^{\text{bind}}$. If binding occurs, *N*_*A*_ is decremented by 1 if a second random number $R_{j}^{2}\in \left[0,1\right)$ is smaller than $k_{1}N_{A}∕\left(k_{1}N_{A}+k_{3}N_{A^{*}}\right)$; otherwise $N_{A^{*}}$ is decremented by 1. An analogous scheme with independent random numbers is used for unbinding. If a synapse is already bound, it releases the stabilizing entity with probability $p_{j}^{\text{unbind}}=\left(k_{2}^{j}+k_{4}^{j}\right)\Delta t$ where $k_{2}^{j}$ and $k_{4}^{j}$ depend upon the stimulation $I_{j}^{A}$ according to Equation 1. If unbinding occurs, *N*_*A*_ is incremented by 1 with probability $k_{2}^{j}∕\left(k_{2}^{j}+k_{4}^{j}\right)$; otherwise $N_{A^{*}}$ is incremented by 1.

See Table 1 for a description of all the parameters used.

**Table 1:** **Model parameters**.

**WRITE-PROTECTED MODEL**									
**Neuron model**									
**Membrane**			**Threshold**		**Connections**				
*V*^exc^	0 mV		τ_thr_	2 ms	τ_ampa_		5 ms	τ_adapt_	250 ms
*V*^rest^	−70 mV		ϑ^rest^	−50 mV	τ_nmda_		100 ms	*g*^spike^	10
*V*^inh^	−80 mV		ϑ^spike^	100 mV	β		0.5	*w*_−_	0.05
τ_*m*_	20 ms							*k*_*w*_	3
**Synaptic model**									
**Synaptic state**					**Plasticity induction**				
τ_w_	200 s		*a*_wT∕Tz_	3.5	*A*_−_		2 × 10^−4^	*k*_up_	1 s^−1^
τ_T_	200 s		*a*_Tw_	1.3	*A*_+_		5 × 10^−4^	*k*_down_	1/2000 s^−1^
τ_z_	200 s		*a*_zT_	0.95	τ_*x*_		16.8 ms	θ	0.01
*k*_w_	3		τ_γ_	600 s	τ_*y*_		33.7 ms		
σ	10^−2^ s^−1∕2^		ϑ_γ_	0.37	τ_triplet_		114 ms		
**STATE-BASED MODEL**									
**Transition rates**									
α	0.017 min^−1^	β	0.067 min^−1^	τ_*e*_	0.017 min^−1^	τ_*l*_	0.01 min^−1^	τ_*r*_	1 × 10^−4^ min^−1^
**RECONSOLIDATION MODEL**									
**Full model**								**Reduced model**	
*a*_*b*_	1		*k*_0_	5 × 10^−4^ s^−1^ (wp)	*I*_2_0__		5.7 × 10^−2^ (wp)	φ	72 s
*k*_1_	1/10,000 s^−1^ (wp)			0.02 s^−1^ (sb)			5.8 × 10^−2^ (sb)	ϑ_big_	−80 s^−1^
	1/3,000 s^−1^ (sb)		*m*	2 × 10^−3^ s^−1^ (wp)	*I*_4_0__		4.5 × 10^−2^	η	10 s
*k*_3_	1/1,750 s^−1^ (wp)			0.25 s^−1^ (sb)	*N*_*A, tot*_		2 × 10^4^	ω	5.4 s
	1/30 s^−1^ (sb)		*r*	3 × 10^−3^	τ_*A*_		150 s		

For the reconsolidation extension different values were used for some of the parameters between the write-protected (wp) and state-based (sb) model, as indicated.

### Reduced reconsolidation model

To understand the dynamics of synaptic reconsolidation, we compare simulations of the full stochastic model with an effective model of the stabilizing entities (*A* and A^*^) inside the postsynaptic cell. Besides providing analytical insight into the reservoir dynamics, the reduced model also allows us to rapidly explore the parameter space for cellular reconsolidation so as to trace the “boundary conditions” observed in experimental data. The reduced model is derived as follows: first, we note that it is sufficient to study the mean of the number of stabilizers, *n*_*A*_ = 〈*N*_*A*_〉 and $n_{A^{*}}=\langle N_{A^{*}}\rangle$, because the fluctuations around the mean are not essential for the reconsolidation mechanism. Second, we approximate the synapse specific unbinding rates $k_{2}^{j}$ and $k_{4}^{j}$ by average non-specific rates ${\hat{k}}_{2}$ and ${\hat{k}}_{4}$, respectively. Those non-specific rates still depend upon the input $I^{A}=I_{j}^{A}$ (assumed to be the same for all synapses), but no longer depend upon the synapse index. This allows us to obtain a reduced two-dimensional description for the mean number of stabilized synapses and the mean reservoir size,

(3) $$\begin{array}{l} \frac{d}{dt}n_{A\text{Syn}}=-\left({\hat{k}}_{4}+{\hat{k}}_{2}\right)n_{A\text{Syn}}+(k_{1}n_{A}+k_{3}n_{A^{*}})\\ \;(N_{\text{big}}-n_{A\text{Syn}}) \end{array}$$

(4) $$\frac{d}{dt}n_{A^{*}}\;=\;-k_{3}n_{A^{*}}(N_{\text{big}}-n_{A\text{Syn}})+{\hat{k}}_{4}n_{A\text{Syn}}.$$

Here, $n_{A}=N_{A,\text{tot}}-n_{A\text{Syn}}-n_{A^{*}}$, and $N_{\text{big}}=\sum_{j=1}^{N}H\left(z_{j}\right)$ is the number of big, or consolidated, synapses on the postsynaptic neuron. Equations 3 and 4 are approximations because, in general, ${\hat{k}}_{2}n_{A\text{Syn}}\neq \sum_{j}k_{2}^{j}p_{A\text{Syn}}^{j}$ since there are correlations between the unbinding rate and the probability $p_{A\text{Syn}}^{j}$ of synapse *j* being in the bound state. The same holds true for ${\hat{k}}_{4}$. Numerical simulations verified that these approximations are accurate enough to predict the evolutions of the full model. Equations 3 and 4 correspond to the simplified reaction

(5) $$\begin{array}{l} A+\text{Syn}\overset{k_{1}}{\underset{{\hat{k}}_{2}\left(I^{A}\right)}{⇌}}(A\text{Syn})\overset{{\hat{k}}_{4}\left(I^{A}\right)}{\underset{k_{3}}{⇌}}A^{*}+\text{Syn} \end{array}$$

of *N*_*big*_ synapses with *N*_*A, tot*_ stabilizers. The unbinding rates ${\hat{k}}_{2}$ and ${\hat{k}}_{4}$ are given by Equation 2 with the global activity *I*^*A*^(*t*) modeled as

(6) $$\tau_{A}{\dot{I}}^{A}=-I^{A}+\omega \sum_{k}\delta \left(t-t_{k}\right).$$

Here, ω is a fixed increase that occurs with each pulse of stimulation during LFS captured by the sum of δ functions.

### Dynamics of the reservoir

**Figure 2:**
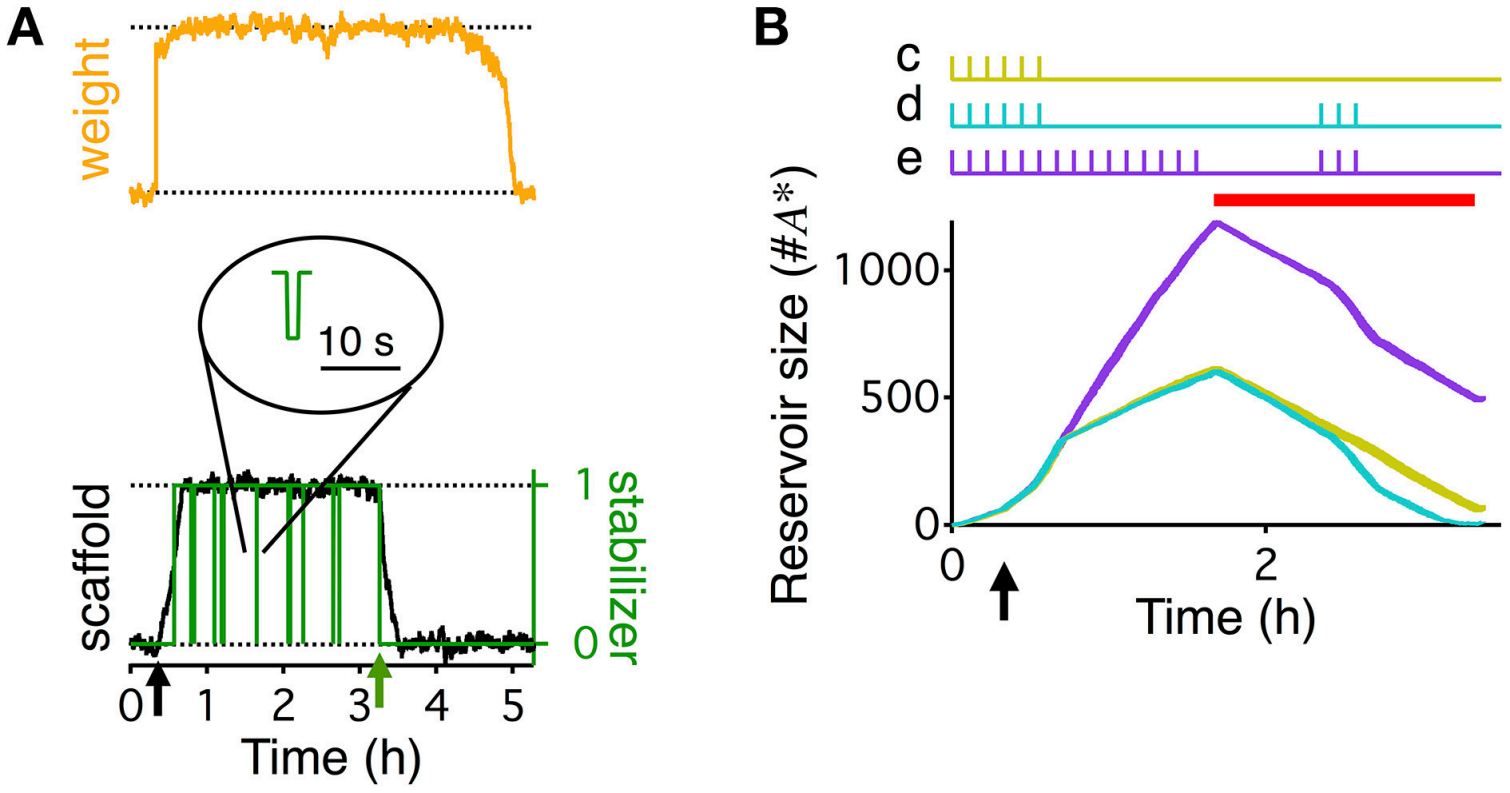
**Stabilization of the consolidated synaptic state and reservoir dynamics. (A)** Weight (orange), scaffold (black), and binding state (green) of a single synapse in the extended write-protected model (see Materials and Methods section). HFS, to induce LTP, occurs at the point indicated by the black arrow. Green line indicates if the synapse is bound by a stabilizing entity (value of 1), or unbound (value of 0). At around 3 h into the simulation (green arrow) the synapse was artificially set to be “unbound” and kept “unbound” thereafter. This destabilizes the scaffold, causing it to degrade to its low state, which then eventually forces the tag and the weight to decay to their low states as well. Inset circle shows the magnified time course of the binding state of the synapse. While the synapse is big, unbinding of a stabilizer is followed, within a few seconds, by the binding of the same or another stabilizing entity. **(B)** Evolution of the reservoir of PSI immune stabilizers in response to different stimulation paradigms (shown at the top), corresponding to Figures 1C–E, indicated by the lower case letters next to the stimulations. The reservoir grows until the initiation of PSI, whose duration is indicated by the red bar. The reservoir grows faster in the presence of activity. Once protein synthesis is inhibited, the reservoir decays, decaying faster in the presence of activity. If during PSI the reservoir drops to zero, consolidation degrades because no stabilizing entities are available to stabilize the scaffolds of the consolidated synapses, similar to the scenario depicted in **(A)**. The time axis is magnified compared to Figure 1 (3 vs. 10 h) to highlight the differences in the reservoir dynamics that underlie the different conditions. The width of the line indicates s.e.m averaged over 10 repeats.

**Figure 3:**
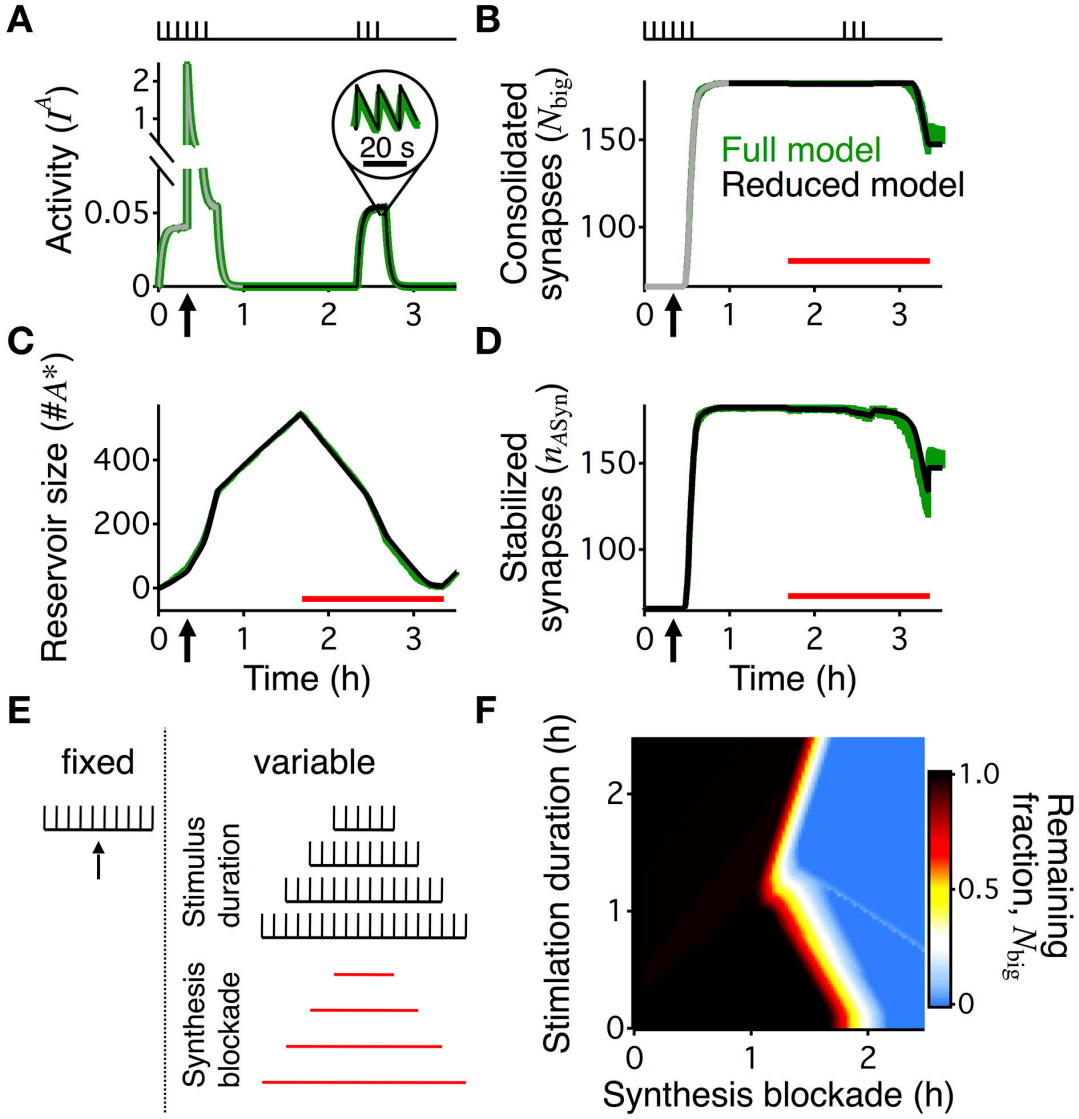
**Boundary conditions for reconsolidation mapped using a reduced model**. The reduced model (black) approximates the activity averaged across all synapses **(A)**, the number of consolidated synapses **(B)**, the dynamics of the reservoir **(C)**, and the number of stabilized synapses **(D)** of the detailed write-protected model (green). The stimulation protocol is shown above the plots for **(A,B)**, a red bar shows the duration and timing of the PSI, and an arrow indicates the time of the HFS. For **(A,B)** the gray line shows the 60 min initiation phase, as simulated with the detailed model. The prediction of the reduced model only begins with the black line after 60 min of simulation. For **(A–D)** the width of the line indicates s.e.m. **(E)** A range of conditions to map out when reconsolidation would and would not occur—the “boundary conditions” to reconsolidation. All simulations began with a period of 40 min of background 0.1 Hz stimulation with a HFS at 20 min to induce LTP, as shown to the left of the dotted line. Arrow indicates the time of the HFS. We varied the duration of the following period with no stimulation and the duration of PSI, with examples shown to the right of the dotted line. Stimulation duration and PSI ranged from 0 s to 2.5 h. Both the stimulation and PSI were centered at 150 min. **(F)** Remaining fraction of consolidated synapses (color code, see scale) at the end of the stimulation protocol relative to the maximal number of consolidated synapses after 60 min for all combinations of different durations of stimulation (vertical axis) and PSI (horizontal axis).

The number $N_{A^{*}}$ of stabilizing entities that is immune to PSI is of particular interest and is called the reservoir in the following. Equations 3 and 4 can be used to quantitatively understand the evolution of the reservoir, as illustrated in Figures 2, 3. For simplicity, we initialize the dynamics with $N_{A^{*}}=0$ for the simulation of the full model, and with $n_{A^{*}}=0$ for the reduced model, such that before LTP induction there are no stabilizing entities in the reservoir yet.

Let us first consider the case with intact protein synthesis. After LTP induction, synapses in the “big” state rapidly bind to a stabilizing entity, since $k_{1}n_{A}>>\left({\hat{k}}_{4}+{\hat{k}}_{2}\right)n_{A\text{Syn}}$ (Equation 3), such that the number of bound synapses *n*_*ASyn*_(*t*) follows the comparatively slow dynamics of *N*_*big*_. More formally, Equation 3 predicts under the stated assumptions *n*_*ASyn*_ = *N*_*big*_. Equation 4 then implies that the reservoir obeys the simple dynamics $dn_{A^{*}}∕dt={\hat{k}}_{4}N_{\text{big}}$, which describes the rising phase of the reservoir in Figures 2B, 3C. In particular, it explains the decrease of the slope after removing the stimulation: in that case ${\hat{k}}_{4}$ goes to its baseline value *k*_0_ so that the increase is slower. Note that the initial increase in the slope is due to the fast rise of the number of consolidated synapses *N*_*big*_ from near zero to its constant maximal value (Figure 3B).

In contrast, in the case of PSI, the pool of non-immune stabilizers degrades immediately. As a consequence, we cannot exactly replace *n*_*ASyn*_(*t*) by the number of consolidated synapses anymore. Setting *n*_*A*_ = 0, Equations 3 and 4 can be combined to $dn_{A^{*}}∕dt=-{\hat{k}}_{2}n_{A\text{Syn}}-dn_{A\text{Syn}}∕dt$. If the reservoir is not yet depleted ($n_{A^{*}}>0$), *n*_*ASyn*_ is roughly constant and we find that the reservoir decays like $dn_{A^{*}}∕dt=-{\hat{k}}_{2}n_{A\text{Syn}}$ (in that case, the number of stabilized synapses *n*_*ASyn*_ is still roughly given by *N*_big_). The activity dependence of ${\hat{k}}_{2}$ thus explains the effect of stimulation on the decrease of the reservoir during PSI (Figure 2B): without stimulations ${\hat{k}}_{2}=k_{0}$, whereas ${\hat{k}}_{2}>k_{0}$ during stimulation leading to a faster decay.

We stated above that with intact protein synthesis Equation 3 lets us deduce that *n*_*ASyn*_ = *N*_big_, i.e., each big synapse is in contact with a stabilizing entity. For rapid changes, however, this is not correct. Moreover, if not enough stabilizers are available not all of the big synapses can be stabilized, and *N*_big_ will itself not be constant. Empirically, the decay of *N*_*big*_ follows that of *n*_*ASyn*_ with a small delay:

(7) $$\begin{array}{l} \frac{dN_{\text{big}}}{dt}=\frac{\Delta n_{A\text{Syn}}(t-\varphi )}{\Delta t}H\left(\vartheta_{\text{big}}-\frac{\Delta n_{A\text{Syn}}(t-\varphi )}{\Delta t}\right), \end{array}$$

where φ is the temporal delay. The negative change in *n*_*A*Syn_ has to be steeper than a (negative) threshold ϑ_big_ for it to manifest itself in a decay in *N*_big_, and

(8) $$\begin{array}{l} \frac{\Delta n_{A\text{Syn}}(t)}{\Delta t}=\frac{n_{A\text{Syn}}(t+\eta )-n_{A\text{Syn}}(t)}{\eta }, \end{array}$$

where η is a time window over which the change in *n*_*A*Syn_ is computed.

The numerical values of φ and η were determined empirically. See Table 1 for a description of all the parameters used.

### Neuron model

A postsynaptic neuron is connected to a random number of presynaptic Poisson neurons drawn from a binomial distribution with a mean of 200. The variability in the number of presynaptic neurons is included to ensure that our results were not dependent upon an exact number of presynaptic neurons, and can occur over a range of connectivity.

The postsynaptic neuron is modeled as a leaky integrate-and-fire (LIF) neuron with conductance based synapses and adaptation (Gerstner et al., 2014). The membrane potential of the LIF neuron evolves according to the equation:

(9) $$\begin{array}{l} \tau_{m}\frac{dV}{dt}\;=\;\;\left(V^{\text{rest}}-V\right)+\;g^{\text{exc}}(t)\left(V^{\text{exc}}-V\right)\\ \;+\;g^{\text{adapt}}(t)\left(V^{\text{adapt}}-V\right) \end{array}$$

where *g*^exc^ and *V*^exc^ (*g*^adapt^ and *V*^adapt^) are the excitatory (adapting) conductance and reversal potential respectively. A spike is emitted when the potential reaches the threshold ϑ. After a spike, *V* is reset to *V*^rest^ and ϑ is set to ϑ^spike^ to implement refractoriness. The threshold then relaxes back to its rest value according to

(10) $$\begin{array}{l} \tau_{\text{thr}}\frac{d\vartheta }{dt}=\vartheta^{\text{rest}}-\vartheta . \end{array}$$

The excitatory conductance, *g*^exc^, has a fast and a slow component, representing α-amino-3-hydroxy-5-methyl-4-isoxazolepropionic acid (AMPA) conductance, and N-methyl-D-aspartate (NMDA) conductance, respectively. The two components are combined as *g*^exc^ = β*g*^ampa^+(1 − β)*g*^nmda^, where β is the relative contribution of AMPA. The time course of the conductance of the AMPA receptor channel is given a by a first order low-pass filter

(11) $$\frac{dg^{\text{ampa}}}{dt}=-\frac{g^{\text{ampa}}}{\tau_{\text{ampa}}}+\sum_{j}\Delta g_{j}S_{j}\left(t\right),$$

where Δ*g*_*j*_ is the plastic synaptic weight of the connection from presynaptic neuron *j*, and *S*_*j*_(*t*) is the spike train arriving from the presynaptic neuron defined as $S_{j}\left(t\right)=\sum_{k}\delta \left(t-t_{j}^{k}\right)$, with $t_{j}^{k}$ being the *k*^th^ spike from the presynaptic neuron *j* and δ the Dirac δ-function. The NMDA receptor channel undergoes a second-order low-pass filtering, such that

(12) $$\begin{array}{l} \tau_{\text{nmda}}\frac{dg^{\text{nmda}}}{dt}=-g^{\text{nmda}}+g^{\text{ampa}}. \end{array}$$

The voltage dependence of NMDA receptors is neglected for the sake of computational efficiency.

For the simulations presented here, there are no inhibitory presynaptic neurons, and therefore no γ-amionbutyric acid (GABA) receptor simulation. However, the postsynaptic neuron has an adaptive component with spike-triggered self inhibition, where the adaptation conductance increases by an amount, *g*^spike^, with each spike of the postsynaptic neuron, otherwise relaxing exponentially to zero as described by

(13) $$\begin{array}{l} \frac{dg^{\text{adapt}}}{dt}=-\frac{g^{\text{adapt}}}{\tau_{\text{adapt}}}+g^{\text{spike}}S(t), \end{array}$$

where $S\left(t\right)=\sum_{k}\delta \left(t-t^{k}\right)$ is the spike train of the postsynaptic neuron.

The initial conditions for the neuron model were: *V*(0) = *V*^*rest*^, ϑ(0) = ϑ^*rest*^, and all conductances (*g*^ampa^(0), *g*^nmda^(0), and *g*^adapt^(0)) were initialized to 0. See Table 1 for a description of all the parameters used.

### Stimulation protocols

The low frequency stimulation (LFS) is modeled by prescribed spiking activity of the presynaptic neurons. During LFS the presynaptic spike trains are jittered versions of a periodic spike train with rate 0.1 Hz, meaning that a spike shifts around the periodic time *t*_0_ by a delay drawn from a Gaussian distribution with mean of 0 and a standard deviation of 3 ms. HFS occurs in the same manner as LFS, but the frequency of stimulation is 100 Hz for 60 s.

### Extended write-protected model

We extend the model of Ziegler et al. (2015) to incorporate reconsolidation. In that model a set of 3 interacting bistable equations governs the state of each synapse. All three variables follow the same bistable dynamics $\tau_{x}ẋ=f\left(x\right)=-\frac{dU}{dx}$ with $U\left(x\right)=\frac{x^{4}}{4}-\frac{x^{2}}{2}$. This equation has two stable fixed points, *x* = +1 and *x* = −1. The first equation determines the weight of the synapse,

(14) $$\begin{array}{l} \frac{d}{dt}w_{j}=\frac{1}{\tau_{w}}f(w_{j})+\frac{a_{Tw}}{4\tau_{w}}(1-G_{j}(t))(T_{j}-w_{j})+\sigma \xi_{j}^{w}(t)+I_{j}^{w}. \end{array}$$

The external input $I_{j}^{w}$, defined below in Equation 23, has units of a stimulation frequency (1∕*s*). The synaptic conductance is determined by Δ*g*_*j*_ = *g*_0_[*w*_−_ + (*w*_*j*_ + 1)(*w*_+_−*w*_−_)∕2], where *w*_+_ = *k*_*w*_*w*_−_. The second equation determines the state of the tag at the synapse,

(15) $$\begin{array}{l} \frac{d}{dt}T_{j}\;=\;\frac{1}{\tau_{T}}f(T_{j})+\frac{a_{wT}}{4\tau_{T}}G_{j}(t)(w_{j}-T_{j})\\ \;+\frac{a_{zT}}{4\tau_{T}}(1-p(t))(z_{j}-T_{j})+\sigma \xi_{j}^{T}(t). \end{array}$$

And the third equation determines the state of the scaffold of the synapse,

(16) $$\begin{array}{l} \frac{d}{dt}z_{j}=\frac{1}{\tau_{z}}f(z_{j})+\frac{a_{Tz}}{4\tau_{z}}p(t)(T_{j}-z_{j})+\frac{a_{b}}{\tau_{z}}H(z_{j})(b_{j}-1)+\sigma \xi_{j}^{z}(t). \end{array}$$

For all of these equations *f*(*x*) = −*x*(*x*−1)(*x*+1), which is the derivative of −*U*, and the terms ξ_*j*_(*t*) are independent Gaussian white noise processes with the properties $\langle \xi_{j}^{\alpha }(t)\rangle =0$ and

(17) $$\langle \xi_{j}^{\alpha }(t)\xi_{i}^{\beta }\left(t^{\prime }\right)\rangle \begin{array}{l} =\delta \left(t-t^{\prime }\right)\;\cdot \;\delta^{\alpha \beta }\;\cdot \;\delta_{ij}. \end{array}$$

The coupling parameter terms *a*_*Tw*_, *a*_*wT*_, *a*_*zT*_, and *a*_*Tz*_ determine the strength of the interactions between the variables. The gating variable *G*_*j*_ couples the weight and the tag, and depends upon a low-pass filtered version of the plasticity-induction stimulus, $I_{j}^{\gamma }$ (with units 1∕*s* as described below in Equation 24), as follows:

(18) $$\begin{array}{ll} G_{j}=H\left(\gamma_{j}-\vartheta_{\gamma }\right),\;\; & \\ \tau_{\gamma }{\dot{\gamma }}_{j}=-\gamma_{j}+\kappa I_{j}^{\gamma }.\;\; & \end{array}$$

That is, *G*_*j*_ switches if γ_*j*_ surpasses the threshold ϑ_γ_. The factor κ = 1*s* takes care of the correct units in the last equation.

The second gating variable *p* couples the tag and the scaffold and represents the availability or concentration of plasticity related products in the postsynaptic neuron. It is dependent upon an external reward or novelty signal, such as dopamine or other neuromodulators, in the following way:

(19) $$\begin{array}{l} \frac{d}{dt}p=D(t)k_{up}(1-p)-k_{down}p. \end{array}$$

The constants *k*_*up*_ and *k*_*down*_ determine the sensitivity of *p* to the dopamine signal *D*(*t*) and the decay rate of *p* in the absence of dopamine, respectively. The dopamine signal in our model is instantaneously switched from 0 to 1 for 60 s, coincident with a HFS stimulation used to induce LTP.

The input dependent terms $I_{j}^{w}$ and $I_{j}^{\gamma }$ are based upon the same standard Hebbian learning rule, but differ in their details. The triplet spike-timing dependent plasticity (STDP) rule with the original set of reduced parameters (Pfister and Gerstner, 2006) was used to characterize Hebbian learning. In the triplet rule, LTP induction

(20) $$\begin{array}{l} I_{\text{triplet}}^{+}=A^{+}x_{j}^{+}(t)y^{t\text{riplet}}(t-\varepsilon )S(t) \end{array}$$

is driven at the moment of postsynaptic spiking, *S*(*t*), and proportional to the two ‘traces’ $x_{j}^{+}\left(t\right)$ and $y_{j}^{\text{triplet}}\left(t\right)$ left by earlier pre- or postsynaptic spikes. ε is an infinitesimally small time step. LTD induction

(21) $$\begin{array}{l} I_{\text{triplet}}^{-}=A^{-}y^{-}(t)S_{j}(t) \end{array}$$

is independent from LTP inductions and occurs in the model at the moment of presynaptic spikes *S*_*j*_(*t*) and proportional to trace $y_{i}^{-}\left(t\right)$ left by earlier postsynaptic spikes. The traces are given by

(22) $$\begin{array}{l} \frac{d\xi_{k}^{\alpha }}{dt}=-\frac{\xi_{k}^{\alpha }}{\tau_{\alpha }}+S_{k}(t), \end{array}$$

where ξ^α^ ∈ {*x*^+^, *y*^triplet^, *y*^−^} and τ_α_ is the respective decay constant. Using the above formulation of the triplet rule as the foundation (Ziegler et al., 2015), the drive for weight induction is

(23) $$\begin{array}{l} I_{j}^{w}\;=\;I_{\text{triplet}}^{+}\left(1+{\left[z_{j}-w_{j}\right]}_{+}\right)\left(1-w_{j}\right)\\ \;-\;I_{\text{triplet}}^{-}\left(1+{\left[w_{j}-z_{j}\right]}_{+}\right)\left(1+w_{j}\right), \end{array}$$

where [*x*]_+_ = *xH*(*x*) denotes linear rectification. The input dependence of the coupling between weight and tag-related variables is

(24) $$\begin{array}{l} I_{j}^{\gamma }=\left[I_{\text{triplet}}^{+}\;\cdot \;H(w_{j}-z_{j})+I_{\text{triplet}}^{-}\;\cdot \;H(z_{j}-w_{j})\right](1-\gamma_{j}). \end{array}$$

In contrast to the original model by Ziegler et al., the equation for the consolidation variable *z*_*j*_, Equation 16, includes an additional coupling term with strength *a*_*b*_ ≥ 0. This term destabilizes the consolidated state when the synapse is not bound to a stabilizing entity. To that end, we introduced a binary variable *b*_*j*_ that is unity if the synapse is bound and zero if it is unbound. If *b*_*j*_ = 1 the novel term vanishes and Equation 16 becomes identical to that in Ziegler et al. (2015). However, if *b*_*j*_ = 0 the novel term tilts the potential to the left such that the right potential well (corresponding to the consolidated state) becomes shallower or even vanishes, thereby destabilizing the consolidation. The Heaviside function in Equation 16 ensures that the destabilization is only effective when the synapse is in the consolidated state.

Transitions between *b*_*j*_ = 0 and *b*_*j*_ = 1 (binding and unbinding) occur according to the kinetics given by Equation 2. The transition rates for unbinding $k_{2}^{j}$ and $k_{4}^{j}$ (Equation 1) depend on the co-activity of pre- and postsynaptic neurons $I_{j}^{A}$. In our model this co-activity is based upon the triplet STDP rule (see Equations 20 and 21, above), such that

(25) $$\begin{array}{l} \tau_{A}\frac{dI_{j}^{A}}{dt}=-I_{j}^{A}+I_{\text{triplet}}^{+}+I_{\text{triplet}}^{-}, \end{array}$$

where τ_*A*_ is the decay constant.

The delayed decay of consolidation discussed in the last paragraphs of the “Results” section, is achieved by an activity-dependent intensity of the noise associated with the weight (Equations 14 and 15) as follows:

(26) $$\begin{array}{l} \frac{d}{dt}w_{j}\;=\;\frac{1}{\tau_{w}}f(w_{j})+\frac{a_{Tw}}{4\tau_{w}}(1-G_{j}(t))(T_{j}-w_{j})\\ \;+\;H\left(I_{j}^{A}-\theta \right)\sigma \xi_{j}^{w}(t)+I_{j}^{w} \end{array}$$

(27) $$\begin{array}{l} \frac{d}{dt}T_{j}\;=\;\frac{1}{\tau_{T}}f(T_{j})+\frac{a_{wT}}{4\tau_{T}}G_{j}(t)(w_{j}-T_{j})\\ \;+\;\frac{a_{zT}}{4\tau_{T}}(1-p(t))(z_{j}-T_{j})+H(I_{j}^{A}-\theta )\sigma \xi_{j}^{T}(t). \end{array}$$

This ensures that the noise is bigger in the presence of activity (as long as the activity is greater than θ).

One third of synapses were initialized to their big, consolidated, state, meaning *w*_*j*_(0) = 1, *T*_*j*_(0) = 1, and *z*_*j*_(0) = 1, the remaining synapses were initialized as unconsolidated, meaning *w*_*j*_(0) = −1, *T*_*j*_(0) = −1, and *z*_*j*_(0) = −1. All of the synapses that were initially consolidated were initialized as bound by stabilizing entities (i.e., initialized in the *A*Syn state). In the initial state γ_*j*_(0) = 0, and all of the STDP parameters ($x_{j}^{+}\left(0\right)$, $y_{j}^{\text{triplet}}$, and *y*^−^(0)) were initialized to 0. See Table 1 for a description of all the parameters used.

### Extended state based model

**Figure 4:**
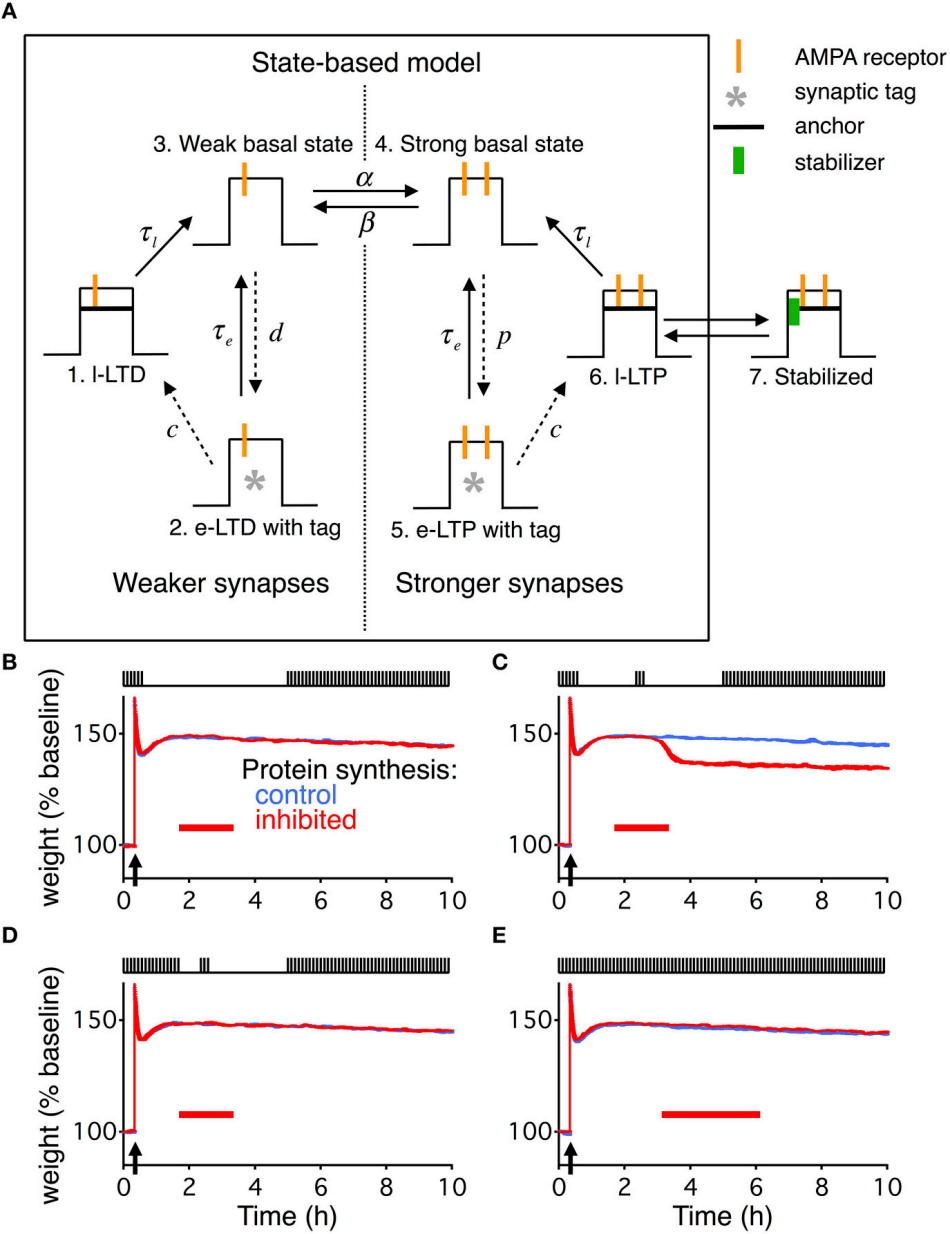
**State-based model extended with dynamic stabilization captures reconsolidation. (A)** The state-based model describes each synapse as being in one of six states, three of which describe weak weights, and three of which describe strong weights. The transitions between the states can either be constant (solid arrows) or dynamic (dashed arrows). The dynamics of the transitions change during a given stimulation paradigm to cause different forms of LTP or LTD. To extend the model we added a 7th state. State 6 and 7 are in dynamic equilibrium with the binding and unbinding of a stabilizing entity. For the extended model the transition from state 6 to state 3 is relatively fast, while state 7 has a very slow decay time. **(B–E)** are identical conditions as in Figures 1C–F, with the same layout, and the width of the line indicates s.e.m averaged over 10 repeats.

We extend the model of Barrett et al. (2009) to capture reconsolidation. Each synapse exists in one of 7 different states (the original model only had 6 states). States 1–3 correspond, with differing amounts of stability, to a weak synapse, while states 4–7 correspond, with differing amounts of stability, to a strong synapse (see Figure 4A). We assume 1000 synapses in the model. Initially 800 are in the weak state and 200 are in the strong state. The relative field excitatory post-synaptic potential is

(28) $$\%\text{ fEPSP }\left(t\right)=\frac{1}{1200}\left(\sum_{i=1}^{3}N_{i}\left(t\right)+\sum_{i=4}^{6}2N_{i}\left(t\right)\right)\;\times \;100\%,$$

where *N*_*i*_(*t*) are the number of synapses that occupy state *i* at time *t*. Because the strong states carry twice the weight of the weak states, the scaling factor $\frac{1}{1200}$ is due to the fact that initially 80% of the 1000 synapses are weak, so that the division by 800 × 1 + 200 × 2 ensures an initial value of 100%.

The state-based model works through the changes in the transition rates between the seven states in response to different stimulus paradigms, such as HFS to cause LTP, or LFS to cause long-term depression (LTD). In the absence of any LTP or LTD inducing stimulus, the weak and strong basal states (states 3 and 4) transition back and forth by a rate of α (weak to strong) and β (strong to weak). The intermediately stable weak and strong states (states 2 and 5) both transition back to the basal states with a fixed rate of τ_*e*_, and the stable weak and strong states (states 1 and 6) both transition to the basal states with a fixed rate of τ_*l*_.

The original paper (Barrett et al., 2009) used one burst of HFS to induce early LTP, and 3 bursts of HFS to induce consolidation and late LTP. Here we follow the experimental paradigm of Fonseca et al. (2006a), and only use a single burst of HFS for consolidation and to induce late LTP.

Following HFS, α becomes instantaneously very large ($\alpha \left(t\right)=1\text{h}{\text{r}}^{-1}+\delta \left(t-t_{0}\right)$ for stimulation at *t* = *t*_0_), moving all synapses that are in the weak basal state to the strong basal state. The rate from the strong basal state (state 4) to the intermediately stable strong state (state 5) becomes non-zero, following the time course:

(29) $$\begin{array}{l} p(t)=\frac{t-t_{0}}{50\min}\exp\{1-(t-t_{0})/10\min\}\min^{-1}. \end{array}$$

And the transition rate from the intermediately stable strong state (state 5) to the stable strong state (state 6) becomes non-zero, following the time course:

(30) $$\begin{array}{l} c(t)=\frac{t-t_{0}}{30\min}\exp\{1-(t-t_{0})/30\min\;\}\min^{-1}. \end{array}$$

The induction of LTD follows a similar pattern, just in response to LFS; however, that part of the model was not relevant to this study.

The transition from state 6 to 7 occurs through the binding of a stabilizer, and the transition back from state 7 to 6 occurs through the unbinding of a stabilizer. The dynamics of the stabilizer and its binding and unbinding was identical to that described for the extended write protected model, just with different values for some of the parameters. We selected a faster decay rate for the transition from state 6 to state 4, τ_*l*_, making state 6 far less stable than in the original model. State 7 now has the longest decay rate, τ_*r*_, making it the most stable state.

Since the state based model does not have any activity dependence, we used the averaged input values *I*^*A*^ from the reduced model, see Equation 25, at each point in time to determine the unbinding rates.

See Table 1 for a description of all the parameters used.

### Simulation protocols

All simulations were run in Igor Pro (WaveMetrics). The leaky integrate-and-fire neuron for the write protected model was simulated with a time-step of 0.1 ms, the synapse model with the reconsolidation extension was simulated with a time step of 100 ms, and the state based model was simulated with a time step of 1 s. We used the Euler method for the integration of the deterministic neuron dynamics (Sections Neuron Model and Stimulation Protocols), and we used the Euler-Maruyama method for the integration of the stochastic differential equations in Section Extended Write-Protected Model.

All of the code for the simulations has been placed in a github repository, which can be found at https://github.com/dbkastner/SynReconModel.git.

## Results

To model synaptic reconsolidation we took advantage of the finding that the molecular machinery for reconsolidation is distinct from that of consolidation (Taubenfeld et al., 2001; Lee et al., 2004; Li et al., 2013). That allowed us to build upon preexisting models for consolidation, rather than to start from scratch and model both synaptic consolidation and reconsolidation. Existing models of synaptic consolidation (Fusi et al., 2005; Brader et al., 2007; Clopath et al., 2008; Barrett et al., 2009; Ziegler et al., 2015) all describe layers of processes with different time constants. The slowest of these time constants is linked to consolidation. For example a recently described model of synaptic consolidation—the write-protected model for consolidation (Ziegler et al., 2015)—simulates three interacting variables that describe the state of each synapse. The three variables relate to the weight, the tag, and the scaffold of each synapse (Figure 1A), in line with the synaptic tagging and capture hypothesis of consolidation (Redondo and Morris, 2011). A synapse with a “big” scaffold supports a large synaptic weight via the placement of multiple AMPA receptors, and is therefore interpreted as a “consolidated” synapse.

Experimentally, reconsolidation paradigms involve PSI combined with activation of synapses. The synaptic activation during reconsolidation paradigms is significantly weaker than during an LTD protocol. However, once synapses in the write-protected model reside in the consolidated state, the only way to transition back to the unconsolidated state is in response to a stimulation protocol for LTD. Therefore, this model has no capacity to undergo reconsolidation since no amount of PSI combined with activity that is insufficient to cause LTD would cause its synapses to decay from a consolidated state. The same observation holds for other models of consolidation (Fusi et al., 2005; Brader et al., 2007; Clopath et al., 2008; Barrett et al., 2009), none of which can capture reconsolidation, i.e., the degradation of consolidation due to the combined action of PSI and weak synaptic activity.

### Extended write-protected models captures reconsolidation

We analyzed the results of Fonseca et al. (2006a) to determine the essential components for a model of slice-based synaptic reconsolidation. We found two necessary features: stabilization of consolidated synapses, and an activity-dependent reservoir of stabilizing entities that is immune to PSI. We implemented these two features by positing a large pool of stabilizing entities inside the postsynaptic cell, which are available to stabilize all of the cell's consolidated synapses. A stabilizing entity can reside in one of three states (see Materials and Methods, Equation 2): it can either be bound to an individual synapse, leading to the stabilization of that synapse, or it can be in one of two distinct unbound states. In the naïve unbound state, the number of available stabilizing entities is maintained by protein synthesis and decays rapidly during application of PSI. Therefore, the stabilization of a synapse becomes protein-synthesis dependent. Stabilizing entities that are in the modified unbound state, however, can bind to a strong synapse and stabilize it even in the presence of PSI. Thus, unbound stabilizers form two distinct pools—a first pool that can be emptied by PSI, and a second pool where entities are immune to PSI. In the following, the latter will be referred to as the reservoir of immune entities, or simply the reservoir (Figure 1B). Binding and unbinding rates characterize the transitions between the three different states of stabilizing entities. The rates for binding are high and independent of activity, while the rates for unbinding (destabilization) are low and activity dependent.

This simple model, combined with the write-protected consolidation model reproduces the results of Fonseca et al. (2006a), capturing slice-based reconsolidation in four different stimulation paradigms (Figures 1C–F). The first two resemble the initial description of the behavioral manifestation of reconsolidation (Nader et al., 2000). For all paradigms, synapses were stimulated with a low frequency stimulation of 0.1 Hz, as was done in the original set of experiments to monitor the synaptic weights. Consolidation occurred in response to HFS to induce LTP. Following HFS, the low frequency stimulation continued for a brief time before stimulation ceased. After 60 min without stimulation, PSI began. In the first paradigm, no stimulation occurred during PSI (Figure 1C), while in the second paradigm a period (20 min) of low frequency stimulation occurred in the midst of the PSI (Figure 1D). Then in both paradigms there was a time of no stimulation followed by a final extended period of low frequency stimulation.

In response to the first paradigm, where PSI occurred alone without any stimulation, consolidation persisted in the model throughout the entire duration of the experiment, and was indistinguishable from the control condition without PSI (Figure 1C). However, in the second paradigm, where stimulation occurred during PSI, consolidation degraded (Figure 1D). The degradation occurred with a delay and was highly significant compared to control (*p* < 0.005).

The stimulation for the third paradigm was almost identical to that of the second paradigm except that the initial low frequency stimulation continued for more than 1 h after LTP induction. Consolidation persisted in response to this paradigm with and without PSI (Figure 1E).

Finally, the fourth paradigm was the simplest, and most comparable to standard experiments of slice-based consolidation. This paradigm had continuous low frequency stimulation with an extended period of PSI. Consolidation persisted in response to this final paradigm, indistinguishable from the control condition without PSI (Figure 1F).

The extended write-protected model works as follows: the state of each synapse results from the interaction of three bistable systems, which are related to the weight, tag, and scaffold. Once the scaffold transitions from its “small” to its “big” state, it supports the tagging-related and the weight-related variables to also remain in their high state. This high state with “big” scaffold corresponds to the consolidated state of the synapse. In our extension of the model, the scaffold can remain “big” only if it is bound to a stabilizing entity. If the scaffold remains unbound, the consolidated “big” state is unstable and it will jump back to its small state after some time (Figure 2A). As a consequence, the tag and then the weight are in a metastable state, eventually decaying back to their low states as well.

To understand the different behaviors of the model in response to the different stimulus paradigms we need to track the development of the reservoir of PSI immune stabilizing entities (Figure 2B). When protein synthesis is intact the reservoir grows after induction of LTP due to the persistent binding and unbinding of stabilizing entities to “big” synapses. This leads to a net flux of stabilizers into the reservoir. With activity the reservoir grows faster than without activity; hence the longer the low frequency stimulation the larger the reservoir. Once protein synthesis is inhibited, the reservoir decays because some of the immune entities are used for stabilizing those strong synapses that are currently unbound. The decay is faster in the presence of low frequency stimulation because of increased unbinding rates. For a quantitative analysis of the reservoir dynamics, see the Materials and Methods section.

The difference between the responses to the first stimulation paradigm (Figure 1C) and the second (Figure 1D) is that without the stimulation during the PSI the reservoir of immune entities decays more slowly, so that the reservoir of PSI immune entities survives the perturbation and can continue to stabilize all of the consolidated synapses. However, in the presence of stimulation, the reservoir of PSI immune entities decays faster toward zero, so that after a prolonged application of PSI no stabilizing entities remain to maintain all of the scaffolds of the synapses in their big, consolidated states (Figure 2B). This phenomenon depends on the durations of stimulation and PSI. This will be explored in further detail below.

The responses to the second stimulation paradigm (Figure 1D) and the third (Figure 1E) differ in that with the third paradigm there is a larger buildup of the reservoir of PSI immune entities due to the longer initial stimulation such that the reservoir is larger and can withstand the same perturbation during PSI (Figure 2B). This effect is even more pronounced in the case of the fourth stimulation paradigm (Figure 1F) where the reservoir continues to grow during the prolonged stimulation allowing for robustness to the extended protein synthesis blockade even in the presence of continuous stimulation.

### Simplified model maps out the boundary conditions for reconsolidation

The “boundary conditions” for reconsolidation refer to the conditions under which reconsolidation does or does not occur (Eisenberg et al., 2003; Pedreira and Maldonado, 2003; Nader et al., 2005; Morris et al., 2006; Tronson and Taylor, 2007). In order to rapidly explore the boundary conditions of synaptic reconsolidation, we derived a reduced, two-dimensional, description of the reservoir dynamics (see Materials and Methods). This reduced model quantitatively captured the average behavior of the reservoir dynamics, and ultimately the final number of stabilized consolidated synapses at the end of a stimulation protocol. The reduced model also explains the different rates of growth and decay of the reservoir during different phases of the experiment (Figure 2B).

We distinguish two phases of the experiment. First, the rising phase, corresponding to the first 60 min, was simulated with the complete stochastic model. Because during this phase the stimulation was identical for all simulations (20 min @ 0.1 Hz 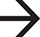 HFS 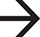 20 min @ 0.1 Hz 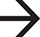 20 min no stimulation) we could use the state of a single detailed simulation at *t* = 60 min as a starting point (initial condition) for multiple runs of the reduced model with different stimulation paradigms. Second, we then used the simplified model to explore the further evolution. In this second phase, all big synapses were initially bound to stabilizers. Decay started when the reservoir of PSI immune entities was empty so that not all big synapses could find a binding partner. In this final decaying phase, the number of big synapses was approximately equal to a delayed version of the number of bound synapses (see Materials and Methods).

The reduced model describes a system of two coupled differential equations for the number of bound synapses and the size of the reservoir of PSI immune entities. Two inputs drive it. First, an activity-related input captures the average joint spiking activity of the pre- and postsynaptic neuron pair (Figure 3A). This activity is approximately given by a low-pass filtered version of the low-frequency stimulation (see Materials and Methods, Equation 8). Second, the number of big synapses (Figure 3B) influences the dynamics because only those synapses can be stabilized, and therefore their number yields the maximal attainable value for the number of bound synapses. We compared the detailed model with the reduced one using a protocol with 20 min of activity centered at 150 min after LTP induction. The reduced model accurately predicted the time course of the reservoir (Figure 3C) and the number of stabilized synapses (Figure 3D) during the entire reconsolidation protocol. In particular, it provided an efficient means to obtain a reliable estimate for the final number of bound synapses, i.e., the number of stabilized synapses at the end of the reconsolidation experiment (Figures 3B,D).

Importantly, the reduced model predicted the presence or absence of synaptic reconsolidation in the full model when tested on 5 arbitrary combinations of the durations of PSI and stimulation (data not shown). This allowed us to explore a broad range of combinations of PSI and stimulation to map out the boundary conditions for reconsolidation. This broad exploration would have taken a prohibitive amount of time if run on the full model, since the full model requires the stochastic simulation of the full neural dynamics including spike generation, and the dynamics of the variables that relate to the weight, tag, and scaffold for every synapse. All simulations began with a period of HFS and then a brief period of background low frequency stimulation. The PSI and stimulation were always centered on the same point in time such that an increase in duration would cause the beginning and ending to occur earlier or later (Figure 3E). Our model of synaptic reconsolidation exhibits a non-monotonic, and relatively complicated boundary conditions to reconsolidation (Figure 3F).

### Extended state-based model also captures reconsolidation

The write-protected model is not the only model of slice-based consolidation. Another useful model is the state-based model (Barrett et al., 2009), which captures diverse features of consolidation. Given the generality of our extension to the write-protected model, we sought to extend the state-based model in a similar way to check the generality of our findings.

The state-based model describes each synapse as residing in one of six states (Figure 4A). The different states correspond to weak or strong synapses with different amounts of stability. The transitions between states are modified based upon the stimulation protocol, such that in response to HFS most synapses end up in a very stable strong state. Like the write-protected model, the state-based model does not have any means for activity dependent degradation of consolidated synapses. Additionally, the state-based model does not have any built-in relationship between how the specific pre- and postsynaptic spiking activity drives synaptic plasticity. All changes result from *ad-hoc* changes in the transition rates.

We extended the state-based model to capture the four stimulation paradigms studied in Fonseca et al. (2006a) (Figures 4B–E) using the identical kinetics developed for the write-protected model. To integrate the dynamic stabilization we added a seventh state (Figure 4A). The transition between the sixth state and the seventh occurs with binding and unbinding of the stabilizing entity, respectively. The sixth state now has a faster decay time, where the seventh state has the slow decay time. Additionally, since the state based model did not have any input dependent activity, we used the activity from the reduced model as an input for the transition rates between binding and unbinding of the stabilizing entity (see Materials and Methods).

### Delayed decay of consolidation requires multiple levels of activity dependence

Both extended models deviated from the results described by Fonseca et al. (2006a) in an informative way. For the second stimulation paradigm (Figures 1D, 4C) where consolidation degrades due to the combination of PSI and coincident stimulation, the average weight decays immediately in both models. However, that is not the case in the slice-based experiment. There, the weight only decays once the stimulus starts up again after the end of the PSI and a prolonged period with no stimulation.

The absence of the delayed decay in our models occurs because once the reservoir is empty (Figure 2B) there can be no dynamic stability of consolidated synapses. In the extended write-protected model without any stabilizing entities the scaffold decays, which then forces the variables related to the tag and the weight to decay (Figure 2A). That decay occurs with only a relatively short delay set by the size of the noise coupled with the time constants on the weight and the tag (Ziegler et al., 2015). In the extended state-based model with no stabilizing entities the synapses can only be in state 6 which has a short decay time to state 3, which then quickly equilibrates with the weak synaptic state (Figure 4A).

**Figure 5:**
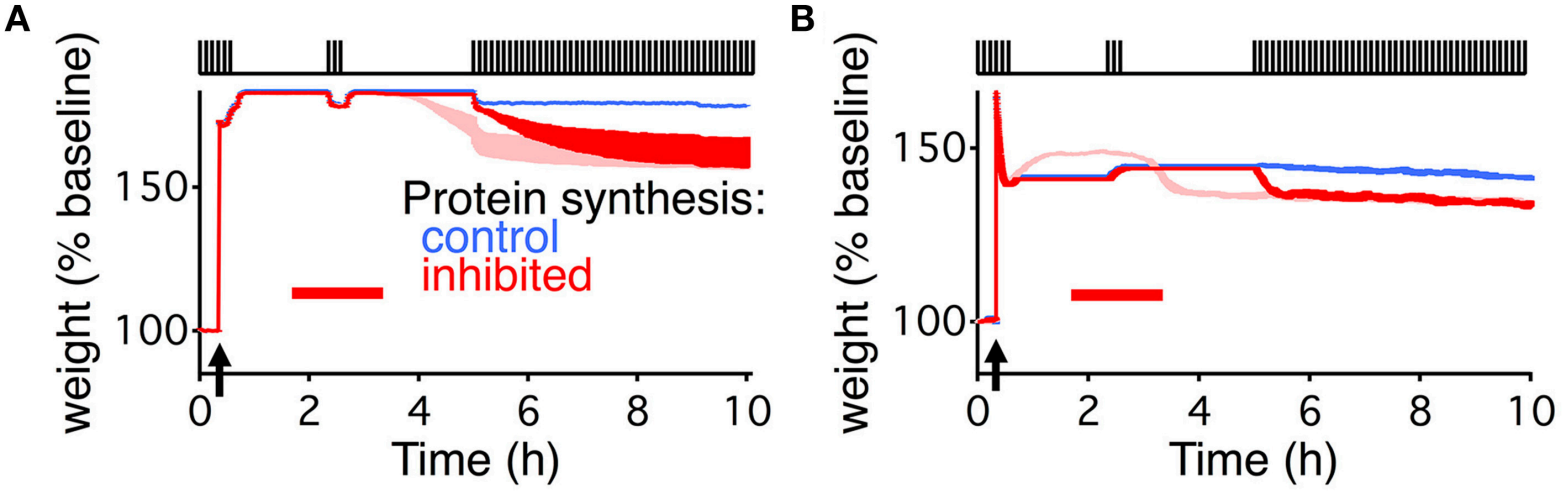
**Delayed decay of consolidation captured by multiple levels of activity dependent processes**. Response of the extended write-protected model **(A)** and extended state-based model **(B)** to the stimulation paradigm shown at the top of the figures, and the same as in Figures 1D, 4C. Arrow indicates the time of the HFS, and red bar shows the duration of the PSI. Blue shows the response of the models with intact protein synthesis, light red shows the response of the original model (reproduced from Figures 1D, 4C). Note that the light red traces begin to decay prior to the beginning of the final stimulation. Red traces show the response of the model with added activity dependent parameters. For the write-protected model the activity dependence is added to the noise of the weight and tag. For the state-based model the activity dependence is added to the transition rate between state 4 and state 3 (β in Figure 4). Note that the red trace and the blue trace are identical until the beginning of the final stimulation. For **(B)** the light and dark red traces differ due to the change in dynamic equilibrium that occurs once β becomes activity dependent. The width of the line indicates s.e.m averaged over 10 repeats.

We were able to capture the delayed decay found by Fonseca et al. (2006a) by adding an additional activity dependence to the two models (Figure 5). For the write-protected model, we used an activity-dependent noise intensity for the bistable dynamics of the weight and the tag, such that in the absence of stimulation the noise vanished. This effectively prevents the weight and the tag from transitioning to the low state before further stimulation (see Materials and Methods). For the state-based model, we added activity dependence to the transition between the weak and strong basal states, such that without stimulation there could be no transition between the states. These additions made the metastable states stable as long as there is no activity.

## Discussion

We have put forward a model that extends two different existing models of consolidation to enable them to capture reconsolidation at the synaptic level. Our model implements, using simple kinetics, two key features of slice-based reconsolidation: stabilization of consolidated synapses through the binding of stabilizing entities (Figure 2A), and an activity dependent reservoir of synaptic stabilizing entities that is immune to PSI (Figures 1B, 2B). By implementing these two features the model reproduces experimental results (Figures 1, 4, 5), capturing conditions where consolidation decays in the presence of PSI coupled with stimulation (Figures 1D, 4C), and conditions where consolidation does not decay either due to no stimulation during application of PSI (Figures 1C, 4B) or due to longer initial stimulation prior to PSI (Figures 1E,F, 4D,E). The model's maintenance or degradation of consolidation rests on the growth and size of its reservoir of PSI immune stabilizing entities (Figure 2B). The reservoir grows and decays in an activity dependent fashion such that the more stimulation occurs prior to PSI and the less stimulation occurs during PSI the greater the robustness of consolidation.

Given the critical nature of the evolution of the reservoir for reconsolidation we developed a reduced two-dimensional model of the reservoir dynamics (Figure 3). This allowed us to explore the boundary conditions of reconsolidation in the model, which revealed a U-shaped boundary for reconsolidation as a function of stimulus duration.

### Model design and limitations

In designing the model, we sought simplicity while still maintaining a connection to the underlying biology. Such biophysically inspired models have proven useful in connecting the molecular and cellular level to the computation performed by neurons and circuits (Ozuysal and Baccus, 2012; David and Shamma, 2013; Ziegler et al., 2015). In such models the goal is not to implement the biophysics of each molecule, even if they were known, but rather to extract core features of the biology to design a simple and informative model of the process under study.

Protein degradation plays a critical role in consolidation (Fonseca et al., 2006b; Karpova et al., 2006; Dong et al., 2008; Cai et al., 2010) and reconsolidation (Lee et al., 2008; Kaang and Choi, 2011; Da Silva et al., 2013). Furthermore, protein degradation is an activity dependent process (Ehlers, 2003; Karpova et al., 2006; Djakovic et al., 2009). Given that we needed to enhance the dynamic capabilities of prior consolidation models we gained inspiration from the biology, and implemented activity dependent unbinding of the stabilizing entity. Although there is no actual degradation of stabilizing entities in our model, the unbinding into the PSI susceptible pool can be viewed as protein degradation, and the dynamics between binding and unbinding can be viewed as the interplay between protein synthesis and protein degradation.

In deciding upon the dynamics of the stabilizing entity, we used the finding that there seems to be limited, shared resources for consolidation (Fonseca et al., 2004). We therefore hypothesized the existence of a finite number of stabilizing entities residing in the postsynaptic neuron, which were shared by all synapses. We chose not to reproduce the finding of competition between synapses to keep the model simple, but with a few additions, such as making binding activity dependent as well, this model probably could display competition between synapses in a situation of reduced protein synthesis.

The existence of a reservoir of immune stabilizing entities seemed a plausible and parsimonious way to capture the experimental results; however, we are not aware of an exact biologic corollary to such a reservoir in this system. Posttranslational modifications, such as phosphorylation, provides enhanced stability for proteins (Cohen, 2000). If reconsolidation relied upon such posttranslational modifications, that would create a reservoir for the stabilization of consolidation. Whether an actual reservoir of posttranslationally modified proteins exists, or if there was just a functional reservoir making use of different types of receptors that are more or less stable at the synapse (Hong et al., 2013), remains to be determined.

In keeping with our goal of simplicity, we chose to have relatively simple dynamics for the binding and unbinding. This limits the model in its robustness to perturbation. For instance, if the duration of PSI were a little longer in Figures 1F, 4E, then consolidation would have decayed. Given the experimental findings, we had no reason to add those complexities, but there remains a possibility for more highly nonlinear or thresholded activity dependent functions. Further slice-based experiments will be helpful in exposing such failings, and help refine the underlying dynamics necessary for reconsolidation. One possible role for more complex dynamics could become relevant when distinguishing the consolidated state from the unconsolidated state. In the current model, the reservoir grows even when the overall neuron has not undergone stable and long-lasting LTP because there are still synapses with big scaffolds. It could very well be that there is a neuromodulatory effect (Lisman et al., 2011) on the size, dynamics, or even existence of the reservoir or PSI susceptible pool to distinguish the global states.

## Author contributions

DK, LZ, and WG designed the research; LZ contributed unpublished reagents; DK and TS performed the research; DK, TS, and WG wrote the paper.

## Funding

This work was supported by the European Research Council (Grant Agreement no. 268689, MultiRules), by a Swiss Government Excellence Scholarship as part of a Fulbright Award (DK) and by NIH R25MH060482 (DK).

### Conflict of interest statement

The authors declare that the research was conducted in the absence of any commercial or financial relationships that could be construed as a potential conflict of interest.
